# A high-throughput SNP discovery strategy for RNA-seq data

**DOI:** 10.1186/s12864-019-5533-4

**Published:** 2019-02-27

**Authors:** Yun Zhao, Ke Wang, Wen-li Wang, Ting-ting Yin, Wei-qi Dong, Chang-jie Xu

**Affiliations:** Zhejiang Provincial Key Laboratory of Horticultural Plant Integrative Biology, Zhejiang University, Zijingang Campus, Hangzhou, China

**Keywords:** Single nucleotide polymorphism (SNP), RNA-seq, Paired-end read length, Trinity, GATK

## Abstract

**Background:**

Single nucleotide polymorphisms (SNP) have been applied as important molecular markers in genetics and breeding studies. The rapid advance of next generation sequencing (NGS) provides a high-throughput means of SNP discovery. However, SNP development is limited by the availability of reliable SNP discovery methods. Especially, the optimum assembler and SNP caller for accurate SNP prediction from next generation sequencing data are not known.

**Results:**

Herein we performed SNP prediction based on RNA-seq data of peach and mandarin peel tissue under a comprehensive comparison of two paired-end read lengths (125 bp and 150 bp), five assemblers (Trinity, IDBA, oases, SOAPdenovo, Trans-abyss) and two SNP callers (GATK and GBS). The predicted SNPs were compared with the authentic SNPs identified via PCR amplification followed by gene cloning and sequencing procedures. A total of 40 and 240 authentic SNPs were presented in five anthocyanin biosynthesis related genes in peach and in nine carotenogenic genes in mandarin. Putative SNPs predicted from the same RNA-seq data with different strategies led to quite divergent results. The rate of false positive SNPs was significantly lower when the paired-end read length was 150 bp compared with 125 bp. Trinity was superior to the other four assemblers and GATK was substantially superior to GBS due to a low rate of missing authentic SNPs. The combination of assembler Trinity, SNP caller GATK, and the paired-end read length 150 bp had the best performance in SNP discovery with 100% accuracy both in peach and in mandarin cases. This strategy was applied to the characterization of SNPs in peach and mandarin transcriptomes.

**Conclusions:**

Through comparison of authentic SNPs obtained by PCR cloning strategy and putative SNPs predicted from different combinations of five assemblers, two SNP callers, and two paired-end read lengths, we provided a reliable and efficient strategy, Trinity-GATK with 150 bp paired-end read length, for SNP discovery from RNA-seq data. This strategy discovered SNP at 100% accuracy in peach and mandarin cases and might be applicable to a wide range of plants and other organisms.

**Electronic supplementary material:**

The online version of this article (10.1186/s12864-019-5533-4) contains supplementary material, which is available to authorized users.

## Background

Single nucleotide polymorphisms (SNPs) are single nucleotide base variations, caused by transitions (C/T or G/A) or transversions (C/G, C/A, or T/A, T/G), in the same position between individual genomic DNA sequences [1, 2]. SNP is the predominant type of DNA polymorphism for genetic variation, which is ubiquitously located in genomes [3, 4] in the intergenic region (regions between genes), coding sequences of genes (exons), or non-coding regions of genes (introns, 5’UTR, 3’UTR, or exon-intron splicing sites) [5]. SNPs in the coding region can be divided into two types, synonymous and nonsynonymous SNPs, with protein sequence affected by the latter type.

Considerable effects on protein function and gene expression can be caused by SNPs occurring in coding regions and regulatory sequences, respectively. Therefore, SNPs are of great potential in genetics, breeding, ecological and evolutionary studies [6]. Due to the high density, scalability and genome-wide distribution, SNPs are considered as ideal genomic resources in genetic studies for the characterization of genetic resources and functional gene identification for traits [7]. In plant genetics for instance, SNPs have been widely used to identify *cis*-regulatory variation within a species based on allele-specific assays and to discover genes linked to complex genetic traits.

With the rapid advances in modern sequencing technology, or next generation sequencing (NGS), de novo and reference-based SNP discovery is performed in numerous organisms, including many plants, even those where there is little or no genetic information available [8]. The availability of NGS provides a convenient approach to discover all SNPs and obtain relevant information on genomic position and genotyping in a single step. Data from reliable large-scale sequencing, especially in diploid plants [9], could improve the cost-effectiveness and efficiency of detection of abundant SNPs. A number of methods have been used for initial SNPs discovery in a high-throughput manner, such as whole-genome sequencing, exome capture, RNA sequencing, methylated DNA sequencing, and restriction enzyme (RE) digestion [10].

Recently, transcriptome sequencing, or RNA-seq, has become one of the most representative high-throughput sequence-based techniques because of its high accuracy and cost-effective. There are multiple advantages in carrying out SNP analysis using RNA-seq data. Firstly, thousands of SNPs can be discovered and the expression levels of functional genes with sequence variations can be observed simultaneously at a reasonable cost. Secondly, the location of variations in coding regions associated with plant biological and agronomical traits can be identified and the phenotypes can be predicted according to genotypes [11]. In addition, it is also a useful platform for related studies like gene characterization, gene expression quantification as well as post translational process analysis [12]. For these reasons, RNA-seq is developing into an extensive application in genetic polymorphisms analysis.

The basic procedures for converting raw data generated from whole genome or transcriptome sequencing into a final SNP result include obtaining raw data from NGS platforms, assembling, and SNP calling to identify SNP between the same unigenes or between different samples from different plant varieties or within one sample when the plant is genetically heterogeneous. Different NGS platforms such as Illumina Genome Analyzer, Roche/454 FLX and ABI SOLiD vary in terms of sensitivity, accuracy, reproducibility and throughput and this means that sequencing data obtained from different platforms has various advantages and limitations [13, 14]. At present, Illumina with good sequencing coverage and read quality, is applied to many plant species for SNP detection by RNA-seq. Currently, assemblers such as Trinity, IDBA, oases, SOAPdenovo and trans-abyss [15, 16] and SNP callers, or variant callers, Genome Analysis Toolkit (GATK) [17], Genotyping-by-sequencing (GBS) [18], SAMtools/BCFtools [19], freebayes [20] and SOAPsnp [21], especially the former two callers, are the most extensively used. RNA-seq can produce massive SNPs at relatively low costs. On the other hand, omissions and errors are major obstacles for SNP identification from RNA-seq data. The availability of multiple choices for different read lengths, assemblers and SNP callers make the SNP analysis even more complicated. It was reported that different read lengths, assemblers and SNP callers all contribute to the accuracy and reliability of the final SNP result [22–25]. Development of existing technologies, especially increased read length, improves the quality of raw sequencing data and significantly reduces missing or erroneous SNPs caused by sequencing and assembling errors [25–27]. Therefore, theoretically a longer read length can produce higher quality raw data and influence downstream analyses [28–31], although no reports about the effects of read length on the accuracy of SNP discovery are available. Selection of a suitable assembler is also critical for SNP detection. For example, Jung’s study on freshwater prawn showed that the results were seriously affected by the assemblers adopted, and an approach to obtain a comprehensive and reliable assembly was necessary [32]. The final SNP results were also affected by the SNP callers applied. For example, SNPs called individually from sequencing data from an *Arabidopsis sup1ros1* ecotype by different tools (GeMS, SAMtools and GATK) led to distinct SNP accuracy rate in You’s study [27]. A study about whole-genome sequencing of dairy cattle also showed that various SNP callers cause significant differences in the number of variants [33]. The SNP array at the whole-genome sequence level in chicken showed that appropriate SNP callers had high values in measures of SNPs especially for those with low allele frequency [24].

At present, the best assembling and SNP calling software combination for achieving the most accurate SNP data on RNA-seq is not known. The access to SNP information on RNA-seq data is a formidable task limited by the availability of reliable SNP discovery methods including assembling and SNP calling pipeline to resolve the problems of genotyping errors and missing data. It is therefore crucial to establish a reliable strategy to overcome the limitations in RNA-seq for downstream SNP analyses.

Woody fruit crops are propagated asexually and therefore have a genetic background of high heterozygosity and maintain a genetic resource of high diversity. In this study, two peach cultivars (‘Hujingmilu’ with deep red appearance and ‘Yulu’ with barely pigmented appearance [34]) and two mandarin cultivars (‘Ponkan’ with orange-reddish appearance and ‘Yellowish-peeled Ponkan’ with yellowish appearance [35]) were used for SNP detection. Since anthocyanins and carotenoids are characteristic pigments for ripe peach and mandarin, a study of the presence of SNPs in pigment biosynthesis genes can contribute to the understanding of the possible mechanisms for differential coloration in ripe fruit peel.

Here transcriptome sequencing of two peach cultivars and two mandarin cultivars was completed and for peach, two paired-end read lengths was involved. The raw data were processed with five common assemblers and two SNP callers and predicted SNPs were generated. The predicted SNPs inside a number of selected genes of peach (genes involved in anthocyanin biosynthesis) and mandarin (genes involved in carotenoid biosynthesis) were compared with authentic ones identified via PCR amplification, gene cloning and sequencing procedures. The effects of different paired-end read lengths, assemblers, and SNP callers on the accuracy of SNP results were investigated and it was found that SNPs can be accurately discovered by performing RNA-seq with a 150 bp read length, assembling with Trinity and SNP calling with GATK. The study provides general guidelines for accurate SNP discovery from transcriptome data.

## Results

### Overview of transcriptome sequencing

Transcriptome sequencing of peach cultivars ‘Hujingmilu’ (‘HJ’) and ‘Yulu’ (‘YL’), and mandarin cultivars ‘Ponkan’ (‘PK’) and ‘Yellowish-peeled Ponkan’ (‘YP’) was performed by Illumina HiSeq™ 2500 and 4000 (the raw data were deposited in NCBI under accession number SRP155137). Paired-end read lengths of 125 bp and 150 bp were applied to peach and 150 bp to mandarin. A summary of the sequencing statistics is shown in Table 1. A total of 40.7 G data, on average 6.7 G data for each library, were obtained. A collection of over 20 million high-quality clean reads were obtained from each library with the Q20 > 92% (Table 1). Quality Score showed the corresponding incorrect base calling in each library was less than 0.01%. The GC dinucleotide content was over 44%. After pre-processing filtering of low-quality sequences and adaptor trimming, high quality sequencing reads that passed thresholds were assembled for further SNP discovery analysis.

**Table 1:** Summary of the sequencing data of ‘Hujingmilu’ (‘HJ’) and ‘Yulu’ (‘YL’) peach and ‘Ponkan’ (‘PK’) and ‘Yellowish-peeled Ponkan’ (‘YP’) mandarin libraries with either 125 bp or 150 bp paired-end read lengths

Samples	Clean Read Number	Base Number	GC Content (%)	N (%)	Q20 (%)	Q30 (%)
HJ-125 bp	22,182,670	5,585,820,171	45.96	0.01	92.64	87.38
YL-125 bp	20,122,397	5,066,451,719	46.06	0.01	92.69	87.41
HJ-150 bp	25,905,927	7,736,134,266	46.14	0.00	95.46	89.74
YL-150 bp	21,836,682	6,522,644,616	46.48	0.00	93.88	86.88
PK-150 bp	29,498,009	8,784,662,004	44.48	0.00	96.74	92.90
YP-150 bp	23,641,003	7,038,555,478	44.34	0.00	96.79	93.00

### De novo assembly and transcriptome annotation

Reads filtered, processed and assembled into contigs as described above were used for SNP discovery. To find an optimum assembly method for SNP detection purposes, the six libraries were analysed using five different de novo assemblers including Trinity, IDBA, oases, SOAPdenovo, Trans-abyss. Due to the different standards of contig length and N50 length applied by the five assemblers, different assembly outcomes were generated. The length of unigenes ranged from 200 bp to 3000 bp for all five assemblers. Taking Trinity as an example, clean reads from three libraries were assembled de novo into 106,574, 103,342, 167,580 transcripts with a mean size of 1545 bp, and 44,470, 49,235, 66,148 unigenes with a mean size of 856 bp, respectively (Additional file 1: Table S1). A summary of the detailed length distribution of transcripts and unigenes is listed in Additional file 1: Table S1.

The alignment results with six libraries using Trinity are shown in Additional file 2: Table S2. Over 76% of total clean reads were mapped to associated unigenes in each library (Additional file 2: Table S2), suggesting that the quality of unigene data was high enough for subsequent SNP calling procedures. When the paired-end read length was 125 bp, the percentages of multi-mapped reads were approximately 60% in both ‘HJ’ and ‘YL’ and that of uniq-mapped reads were around 40% (Additional file 2: Table S2). The percentages of multi-mapped reads declined dramatically to less than 30%, meanwhile, the percentages of uniq-mapped reads were significantly higher when the paired-end read length was 150 bp (Additional file 2: Table S2). These data implied a strong influence of paired-end read length on read mapping quality.

### Single nucleotide polymorphism prediction

**Fig. 1:**
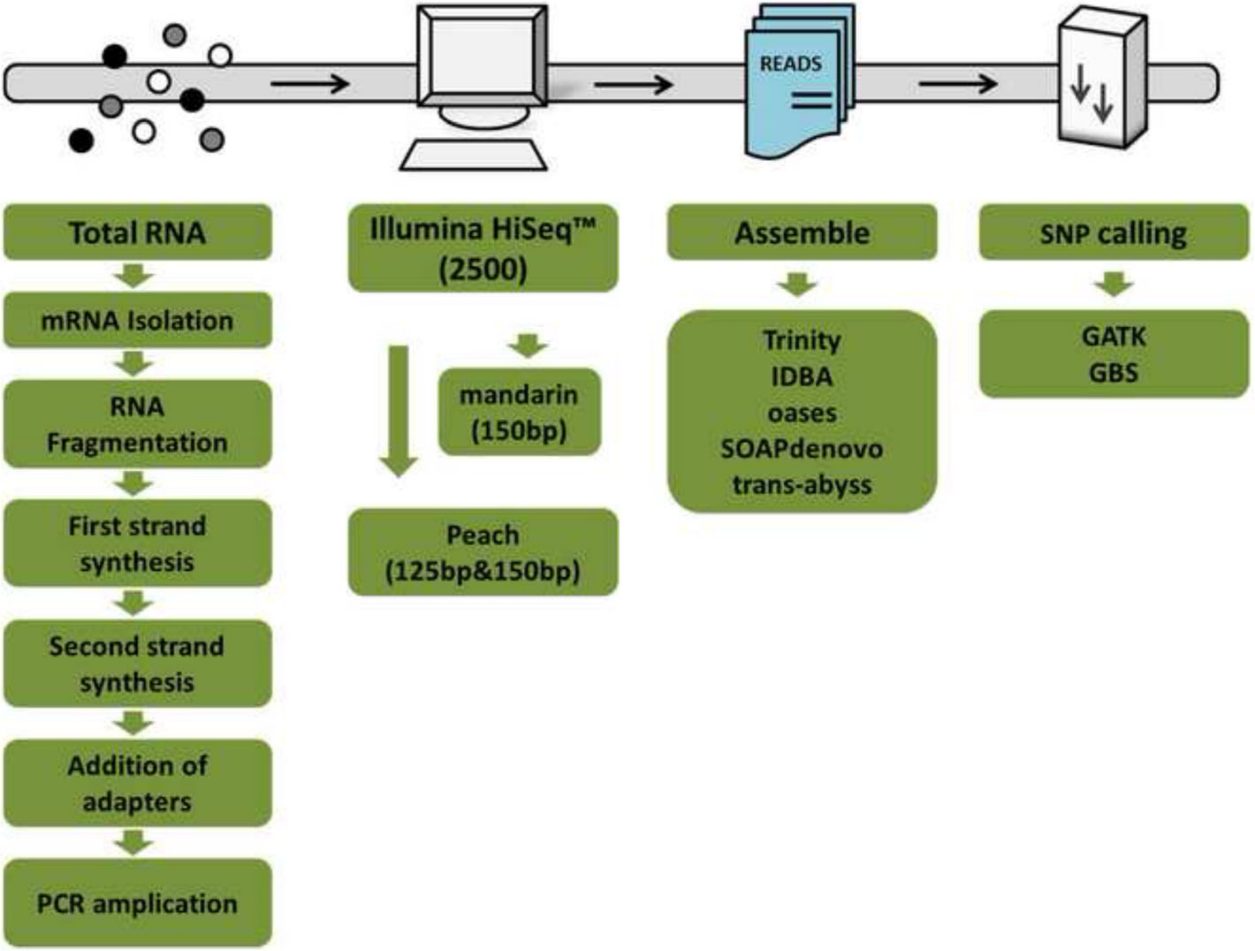
A simplified workflow of analysis strategies for RNA-seq and SNP discovery. The clip arts are drawn with PowerPoint 2010

As illustrated in Fig. 1, two SNP callers (GATK and GBS) were applied to analyse RNA-seq data for SNP discovery following assembly with five different assemblers. We processed SNP calling under parameters described in the Methods section to maximize reliable SNPs and minimize omissions and false-positive SNPs. After calling peach and mandarin transcriptome data with GATK and GBS, predicted SNPs were recorded. Depending on the paired-end read lengths, and the assemblers and SNP callers employed, 7752–56,271 and 43,944–127,201 SNPs were found residing in peach and mandarin transcriptomes, respectively (Additional file 3: Table S3). Comparing data from two SNP calling methods, GATK showed a significantly (1.2–3.5 times) higher number of SNP than GBS in all libraries from five assemblers employed (Additional file 3: Table S3). The data from two paired-end read lengths (125 bp and 150 bp) in peach were compared and it was found that the number of SNP predicted with the same assembler and SNP caller was affected by read lengths. For four assemblers, except for oases, the number of SNPs predicted was higher when the read length was 125 bp (Additional file 3: Table S3). Irrespective of the read lengths and SNP callers employed, the minimum number of SNPs was generated with assembler IDBA_tran for both peach and mandarin (Additional file 3: Table S3).

### Identification and validation of putative SNPs in selected genes

To explore which assembler and SNP caller were most suitable for accurately detecting SNPs, the SNPs predicted through transcriptomic analysis were compared with those obtained via PCR amplification followed by gene cloning and sequencing procedures. Five anthocyanin biosynthesis related genes in peach and nine carotenogenic genes in mandarin possessing putative SNPs as predicted by at least one combination of read length, assembler and SNP caller were selected. The authentic SNPs presented inside these genes were obtained following PCR, gene cloning and sequencing procedures (Additional file 4: Table S4 and Additional file 5: Table S5).

A total of 40 authentic SNPs were identified in the five anthocyanin biosynthesis related genes (CHS, DFR, ANS, UFGT and WD40–1) of peach (Additional file 4: Table S4), and the number of SNPs predicted through different combinations of assemblers and SNP callers for transcriptome analysis as well as the accuracy are summarized in Table 2. Five assemblers and two SNP callers were applied in this study, which made up ten combinations in total. All these combinations were applied to transcriptomic data from paired-end read lengths of 125 bp and 150 bp, respectively, and an overview of raw data for targeted genes listed in Additional file 6: Table S6 and Additional file 7: Table S7.

**Table 2:** Accuracy of SNP predictions from ten combinations of assemblers and SNP callers with 40 authentic SNPs presented in five anthocyanin biosynthesis related genes in peach as example. The RNA-Seq was performed under the read lengths of 125 bp and 150 bp

No.	Paired-end read length (bp)	Assembler	SNP caller	Authentic SNPs presented	True SNPs discovered	True SNPs rate (%)	False positive SNPs predicted	False positive SNPs rate (%)
1	125	Trinity	GATK	40	25	62.50	11	30.56
2	125	Trinity	GBS	40	14	35.00	10	41.67
3	125	IDBA_tran	GATK	40	28	70.00	16	36.36
4	125	IDBA_tran	GBS	40	0	0	0	0
5	125	oases	GATK	40	32	80.00	40	55.56
6	125	oases	GBS	40	3	7.50	9	75.00
7	125	SOAPdenovo	GATK	40	23	57.50	5	17.86
8	125	SOAPdenovo	GBS	40	5	12.50	3	37.50
9	125	Trans-abyss	GATK	40	24	60.00	8	25.00
10	125	Trans-abyss	GBS	40	3	7.50	1	25.00
Average	125	/	/	40	15.70	39.25	10.30	34.45
11	150	Trinity	GATK	40	40	100.00	0	0
12	150	Trinity	GBS	40	6	15.00	0	0
13	150	IDBA_tran	GATK	40	28	70.00	4	12.50
14	150	IDBA_tran	GBS	40	6	15.00	2	25.00
15	150	oases	GATK	40	39	97.50	1	2.50
16	150	oases	GBS	40	6	15.00	0	0
17	150	SOAPdenovo	GATK	40	35	87.50	3	7.89
18	150	SOAPdenovo	GBS	40	6	15.00	0	0
19	150	Trans-abyss	GATK	40	33	82.50	5	13.16
20	150	Trans-abyss	GBS	40	2	5.00	0	0
Average	150	/	/	40	20.10	50.25	1.50	6.11

The accuracy of SNP discovery in peach was compared between ten combinations of assemblers and SNP callers. Different read lengths under the same assemblers and SNP callers resulted in distinct results for the same samples. The accuracy of SNP discovery, represented by the higher percentage of true SNP discovered and a much lower percentage of false positive SNP predicted, was higher with a read length of 150 bp compared to 125 bp (Table 2). For ten combinations, the average percentage of true SNP discovery was 50.25 and 39.25% respectively when the read lengths was 150 bp and 125 bp, while the percentage of false positive SNP discovery was 6.11 and 34.45% (Table 2). Moreover, with a read length of 125 bp, a combination with a higher accuracy was often accompanied by a higher error rate, e.g., Oases-GATK had the highest accuracy of 80.00%, but its error rate was also highest, up to 55.56% (Table 2). There was no obvious correlation between the percentage of authentic SNP discovery and the percentage of false positive SNP discovery with read length 150 bp (Table 2). The accuracy of SNP discovery was also affected by the combinations of assemblers and SNP callers. When the read length was 150 bp, the combination of Trinity and GATK produced the highest accuracy, i.e., 100% discovery of authentic SNP and no prediction of any false positive SNP (Table 2). No combination with 100% accuracy was observed when the read length was 125 bp, further indicating the importance of longer read length. For read length 150 bp, no false positive SNP was predicted with four assemblers (with the single exception of IDBA) when the SNP caller GBS was taken. Compared with GATK, the high filtration standard of GBS reduced the error rate, but on the other side, missed a lot of authentic SNPs and the rate of true SNP discovery dropped to 15% with four assemblers and to 5% with the assembler Trans-abyss (Table 2). For GATK, the accuracy with five assemblers reached 70% or above. However, only the combination Trinity-GATK reached 100% accuracy and no false positive SNP prediction, while IDBA-GATK had the lowest accuracy of 70% and trans-abyss-GATK had the highest false positive SNP rate of 13.16% (Table 2). The study showed that not only SNP callers, but also assemblers had a strong influence on accuracy of SNP discovery from RNA-seq data.

To investigate whether the optimum combination of assembler and SNP caller is independent of plant species, all ten combinations were also applied to transcriptomic data from mandarin with read length of 150 bp. Nine carotenogenic genes (*ZEP*, *PSY1*, *PSY2*, *BCH1*, *BCH3*, *VDE*, *LCYB*, *CYCB*, *CCD1*) were chosen to validate. A total of 240 authentic SNPs were identified, via PCR amplification and gene cloning and sequencing strategy (Additional file 5: Table S5), and the SNPs predicted, via bioinformatic strategy, are listed in Additional file 8: Table S8. A summary of the number of SNPs predicted through different combinations of assemblers and SNP callers for transcriptome analysis as well as the accuracy were summarized in Table 3. Similar to the findings on peach, only the combination of Trinity with GATK produced 100% accuracy in the mandarin case, followed by trans-abyss-GATK with an accuracy rate of 80.42% (Table 3). Among all SNP discovery strategies, the GBS caller always filtered out a lot of true SNPs and the rate of true SNP discovered was substantially inferior to GATK. This is consistent with the findings from peach data analysis. In summary, the combination of Trinity with GATK was the best strategy for SNP discovery, obtaining 100% accuracy in peach and mandarin when the read length was 150 bp, and this strategy might be applicable to wide range of plants and other organisms.

**Table 3:** Accuracy of SNP predictions from ten combinations of assemblers and SNP callers with 240 authentic SNPs presented in nine carotenogenic genes in mandarin as example. The RNA-Seq was performed under the read length of 150 bp

No.	Paired-end read length (bp)	Assembler	SNP caller	Authentic SNPs presented	True SNPs discovered	True SNPs rate (%)	False positive SNPs predicted	False positive SNPs rate (%)
1	150	Trinity	GATK	240	240	100.00	0	0
2	150	Trinity	GBS	240	122	50.83	0	0
3	150	IDBA_tran	GATK	240	32	13.33	2	5.88
4	150	IDBA_tran	GBS	240	25	10.42	1	3.85
5	150	oases	GATK	240	122	50.83	12	8.96
6	150	oases	GBS	240	48	20.00	6	11.11
7	150	SOAPdenovo	GATK	240	129	53.75	7	5.15
8	150	SOAPdenovo	GBS	240	88	36.67	14	13.73
9	150	Trans-abyss	GATK	240	193	80.42	13	6.31
10	150	Trans-abyss	GBS	240	94	39.17	6	6.00
Average	150	/	/	240	109.30	45.54	6.10	6.10

### Characterization of SNPs in peach and mandarin transcriptomes

**Fig. 2:**
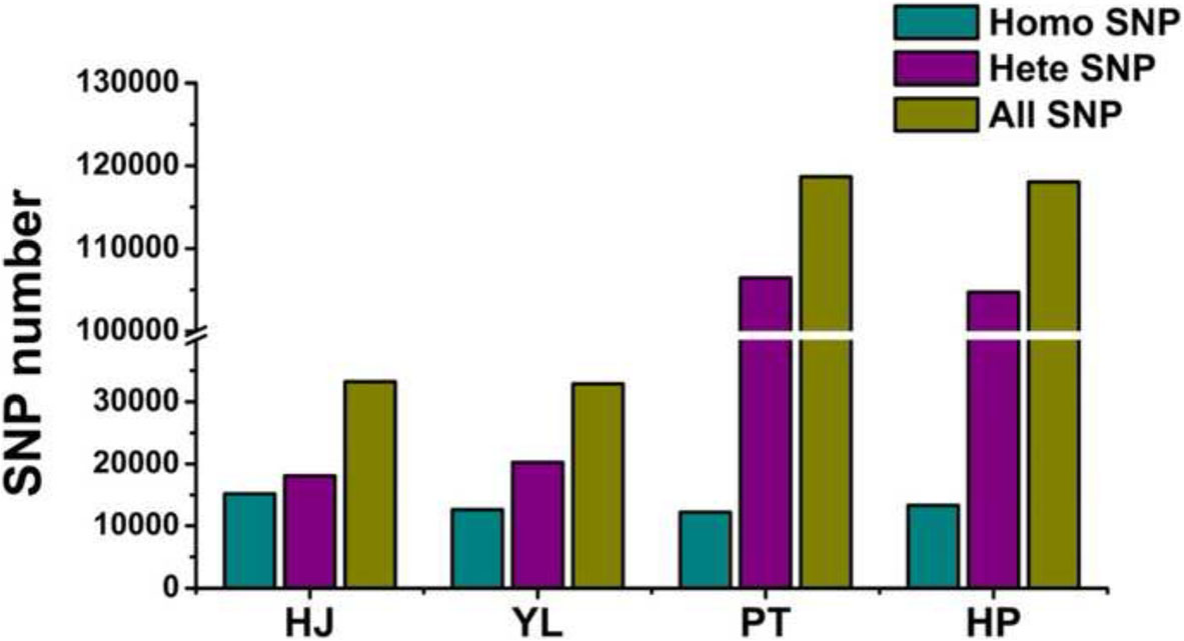
The numbers of heterozygous (purple) and homozygous (cyan) SNPs discovered in peach (cv. HJ and cv. YL) and mandarin (cv. PK and cv. YP) transcriptomes using Trinity and GATK with read length of 150 bp

As described above, using the combination of Trinity with GATK and with a read length of 150 bp, SNPs in transcriptome data of peach and mandarin can be accurately discovered. Here, with such strategy, further characterization of SNPs was performed in the four transcriptomes from two peach cultivars and two mandarin cultivars. The numbers of SNPs predicted is shown in Fig. 2. The number of total SNPs varied only slightly, less than 1.2%, between two cultivars, but was relatively greater between two species, with the number being 2.58 times higher in mandarin than in peach. Overall, the SNP density in mandarin was higher than in peach (Additional file 9: Figure S1).

SNPs can be sorted into two types, including homozygous types for those SNPs presented between cultivars but not between two alleles of an individual cultivar, and heterozygous types for those SNPs presented between two alleles of an individual cultivar. As shown in Fig. 2, the number of heterozygous SNPs is higher than homozygous ones in all four transcriptomes. For all SNPs found in peach cultivars ‘HJ’ and ‘YL’, respectively, 18,072 and 20,248 were heterozygous (HZ) while 15,166 and 12,617 were homozygous (HM), with an average HZ:HM proportion of 1.39:1 in peach. Over three times of SNPs were found in mandarin, compared with peach, and many more heterozygous SNPs (106,466 and 104,732) were found than homozygous SNPs (12,213 and 13,311) with an averaged HZ:HM proportion of 8.29:1 in mandarin (Fig. 2).

For the heterozygous SNPs in all four transcriptomes, fewer transversion substitutions (four possibilities, 38.33 to 40.05%) were found than transitions substitution (two possibilities, 59.95 to 61.67%) (Additional file 10: Figure S2). Transition substitutions significantly outnumbered the transversion substitutions by almost a factor of 2 (Additional file 10: Figure S2). Nearly even distribution between two variation types of transitions (G/A, C/T), and to a lesser extent, between four variation types of transversion (C/A, C/G, T/G, T/A), are observed for the ‘HJ’ transcriptome (Additional file 10: Figure S2). Similar results were found with the other three transcriptomes. Moreover, the distribution patterns of substitution types were also similar between peach and mandarin (Additional file 10: Figure S2), indicating presence of a conserved mechanism during evolution.

## Discussion

RNA-seq dataset and following SNP discovery often differ due to sequencing quality as affected by the read length, sequencing depth and sequencing platforms as well as various downstream analyses [13, 14, 28]. In this study, our data suggested that the accuracy of SNP discovery was affected by paired-end read lengths, assemblers and SNP callers. Previously the effect of read length on accuracy of SNP discovery has not reported, although it was suggested that a higher read length can improve the quality of NGS raw data [31]. Here our data indicated that the longer read length is necessary for high quality SNP discovery. More SNPs were found with 125 bp sequencing, but with higher rate of incorrect prediction compared with 150 bp sequencing, resulting in greatly reduced accuracy with the shorter reads. The reasons for more false positive SNPs predicted from 125 bp sequencing can be attributed mainly to a slightly higher sequencing error and an obviously higher rate of incorrect read mapping. As shown in Table 1, the sequencing error rate (N%) for HJ-125 bp, YL-125 bp was 0.01% while those for HJ-150 bp, YL-150 bp and PK-150 bp YP-150 bp were 0.00%. Additional file 2: Table S2 showed a lower rate of uniq-mapped Reads and a higher rate of multi-mapped Reads for 125 bp sequencing. Mapping to multiple positions can cause erroneous SNPs. Differences between results from different assemblers were observed as well and these can be attributed to the different methods for dealing with sequencing errors, resolving inconsistencies and using paired-end information [32]. Different assemblers vary in the details of usability (ability of installation and execution, speed) and assembly quality (the contiguity and the accuracy), which determine how they resolve errors and inconsistencies in the dataset, leading to distinct assembly performance [14]. Therefore, a suitable assembler is critical in order to improve the quality and performance of de novo assembly. The third factor affecting SNP discovery we found in this study is SNP caller. It was found that each SNP caller has its own characteristics and limitations. For example, although GBS, a stringent SNP caller, can efficiently avoid false SNPs, it easily results in omission because of its limitations such as genotyping errors, missing data and the under-calling of heterozygous sites. The study here revealed the merits and defects of two paired-end read lengths, five assemblers and two SNP callers for SNP analysis, and provide detailed comparison of different methods for reference.

Peach and mandarin are fruit crops propagated asexually and therefore the presence of a high number of heterozygous SNPs (Fig. 2) is not unexpected. The genetic heterogeneity of an organism can be represented by the rate of heterozygous SNP in the genome, and can also, to a large extent, be revealed in the transcriptome as well. The rate of heterozygous SNP among transcriptomes, calculated by dividing the number of heterozygous SNPs with the total length of unigenes (presented in Additional file 2: Table S2), was 0.045 and 0.202% respectively for peach and mandarin. The high rate of heterozygous SNPs in 'PK' and 'YP' mandarins suggested that these two mandarins are not pure mandarins. This is consistent with the recent findings that 'PK' is an early-admixture mandarin containing a small amount of pummelo admixture [36]. Besides, it was found more heterozygous SNPs presented as transition substitutions than transversion substitutions (Additional file 10: Figure S2). This is in consistent with the conclusions that transition mutations, caused by the hypermutability effects of CpG dinucleotide sites and deamination of methylated cytosines, are the most frequent class of SNPs in both plant and animal genomes [37].

## Conclusions

SNP is extensively used as a molecular marker for analysing genotype and trait association as well as assisting breeding. Traditional SNP mining techniques are generally low throughput and technically complicated. High efficient SNP discovery become possible in recent decades due to the availability of huge raw data from next generation sequencing. Although several assemblers and SNP callers have been used in previous literatures, the accuracy was rarely evaluated and the optimum combination of assembler and SNP caller is not clear. In addition, the effect of paired-end read length of sequencing on the accuracy of SNP discovery has not been reported. Here we evaluated the accuracy of SNP discovery from combinations of two paired-end read lengths, five assemblers, and two SNP callers, and established an ideal strategy, i.e., obtaining sequencing raw data from 150 bp paired-end read length, assembling with Trinity and SNP calling with GATK, for SNP discovery. This strategy was proven to be 100% accurate for a total of 280 authentic SNPs analysed, while none of the other strategies reached 100% accuracy. The evaluation was carried out with transcriptome data from peel tissues of two peach cultivars and two mandarin cultivars, but the strategy should also be applicable to other organisms and tissues. With the advantages of high throughput, high accuracy, low cost, and especially, being independent of a reference genome, the method established here can be expected to be widely used for SNP discovery in genetic diversity analysis, breeding and genome-wide association studies.

## Methods

### Plant materials

Ripe fruit and young leaves of ‘Hujingmilu’ (‘HJ’) and ‘Yulu’ (‘YL’) peach [*Prunus persica* (L.) Batsch] as well as ‘Ponkan’ (‘PK’) and ‘Yellowish-peeled Ponkan’ (‘YP’) mandarin (*Citrus reticulata* Blanco) were collected at the Fenghua Peach Research Institute and Quzhou Fruit Science Institute, Zhejiang, China, respectively. All these materials are commercial cultivars with voucher specimens available in the above-mentioned institutes. Peach cultivars ‘YL’ and ‘HJ’ were identified by Yin-chong Zhang in 1883 and A-pan Shao in 1964, respectively [38]. ‘PK’ mandarin is a natural cultivar with a history of over a hundred years and first identifier unknown. ‘YP’ mandarin was identified by Quzhou Bureau of Agriculture Economic Specialty Station in 2008 [35]. The peel tissues, after separation from the fruit, and the young leaves were frozen in liquid nitrogen on the day of collection and stored at − 80 °C for further DNA or RNA extraction.

### DNA and RNA extraction

Young leaves were ground to a fine powder in liquid nitrogen and the genomic DNA was isolated using an improved CTAB method [39]. Peel tissues were also ground to a fine powder in liquid nitrogen and the total RNA was isolated by a CTAB protocol as described by Shan [40] and treated with DNA-free DNA Removal kit (Invitrogen, Life Technologies, CA, USA) to remove DNA contamination. The concentrations of RNA samples were assessed by Qubit (Life Technologies, CA, USA). RNA purity was estimated with the NanoPhotometer® spectrophotometer (IMPLEN, CA, USA) and integrity was evaluated by the RNA Nano 6000 Assay Kit of the Agilent Bioanalyzer 2100 system (Agilent Technologies, CA, USA).

### Library construction and transcriptome sequencing

The library construction and transcriptome sequencing was carried out by staff at Biomarker (Beijing, China). In brief, the library was constructed following mRNA enrichment, mRNA fragmentation, second strand cDNA synthesis, adaptor ligation, size selection, PCR amplification and transcriptome sequencing. The sequencing was performed on an Illumina HiSeq™ 2500 and 4000 platform and paired-end reads were generated. Paired-end read lengths of 125 bp and 150 bp were used for peach cultivars and the length 150 bp for mandarin cultivars. All raw data (raw reads) obtained were further processed through filtering parameters. After removing reads containing adapter or ploy-N and low-quality reads (Q-value ≤10) from raw data, high-quality clean data (clean reads) collected in FASTQ format were used for subsequent analyses. Clean data was further analysed to obtain Q20, Q30, GC content and sequence duplication level for quality control.

### Transcriptome assembly and SNP detection

Five prevalent assemblers, i.e., Trinity, IDBA, oases, SOAPdenovo, Trans-abyss, were applied in all six transcriptome for de novo assembly. All of the generated unigenes were subjected to match against (E-value ≤1.0e-5) public databases (Nr, Swiss-Prot, KEGG, COG, GO) and annotation information was retrieved. The clean reads exported in FASTQ format were aligned to unigenes through STAR software. Subsequently two widely used SNP callers, i.e., GATK and GBS, were applied to perform prediction of SNP site. Reads were processed with a further filtering step under GATK and GBS filter parameters (read depth no less than 10, quality score no less than 20, consecutive single base errors no more than 3 in 35 bp, FS 20.0 window 25). Only SNPs after sequential depth standardization with quality value > 2 were retained. With all programs mentioned, the study resulted in ten different variant calling strategies: Trinity_GATK, IDBA_GATK, oases_GATK, SOAPdenovo_GATK, Trans-abyss_GATK and Trinity_GBS, IDBA_GBS, oases_GBS, SOAPdenovo_GBS, Trans-abyss_GBS. All SNPs were analysed within cultivars and then compared between cultivars. A simplified workflow of assembly and SNP calling is outlined in Fig. 1.

### Identification of SNPs through gene cloning procedures

Genes related to anthocyanin biosynthesis in peach and carotenoid biosynthesis in mandarin were evaluated and the genes containing putative SNP, predicted by at least one variant calling strategy, were chosen for SNP identification through PCR amplification and gene cloning procedures [41]. Briefly, with Faststart High Fidelity PCR system (Roche, SUI), each PCR reaction mixture (25 μL total volume) contained 20 ng genomic DNA, 0.2 μM of each PCR primer (10 μM, Additional file 11: Table S9), 0.1 mM dNTPs, PCR buffer and 0.25 unit of DNA polymerase. PCR was performed with the following cycling conditions: 95 °C for 5 min and 35 cycles of 95 °C for 30s, 58 °C for 30s, and 72 °C for 60s/kb, followed by a final elongation step of 72 °C for 10 min. The PCR products were cloned into pGEM®-T Easy (Promega, USA), and for each gene, at least 15 recombinant plasmids were sequenced and sequence alignment was carried out with Seqman.

## Additional files

Additional file 1: **Table S1.** Assembly statistics of peach and mandarin transcriptomes under Trinity. (DOCX 17 kb)

Additional file 2: **Table S2.** Summary of read mapping of peach (cv. HJ and cv. YL) and mandarin (cv. PK and cv. YP) transcriptomes under Trinity. (DOCX 18 kb)

Additional file 3: **Table S3.** Number of SNPs predicted from RNA-seq data under different paired-end read lengths, assemblers and SNP callers. The number is the average of two cultivars. (DOCX 17 kb)

Additional file 4: **Table S4.** The detailed information of 40 authentic SNPs in five anthocyanin biosynthesis related genes in peach. (DOCX 17 kb)

Additional file 5: **Table S5.** The detailed information of 240 authentic SNPs in nine carotenogenic genes in mandarin. (DOCX 21 kb)

Additional file 6: **Table S6.** An overview of the number of SNPs predicted in targeted genes from peach with ten different strategies under the read length of 125 bp. Values in brackets denote the ratio of heterozygous and homozygous SNP (HZ:HM). (DOCX 19 kb)

Additional file 7: **Table S7.** An overview of the number of SNPs predicted in targeted genes from peach with ten different strategies under the read length of 150 bp. Values in brackets denote the ratio of heterozygous and homozygous SNP (HZ:HM). (DOCX 19 kb)

Additional file 8: **Table S8.** An overview of the number of SNPs predicted in targeted genes from mandarin with ten different strategies under the read length of 150 bp. Values in brackets denote the ratio of heterozygous and homozygous SNP (HZ:HM). (DOCX 22 kb)

Additional file 9: **Figure S1.** SNP density in unigene of peach (cv. HJ and cv. YL) (A) and mandarin (cv. PK and cv. YP) (B) libraries using Trinity and GATK with read length of 150 bp. (JPG 1491 kb)

Additional file 10: **Figure S2.** Classification of heterozygous SNPs predicted in peach (cv. HJ and cv. YL) and mandarin (cv. PK and cv. YP) libraries using Trinity and GATK with read length of 150 bp. (JPG 1763 kb)

Additional file 11: **Table S9.** Primers used for PCR amplification of five anthocyanin biosynthesis related genes in peach and nine carotenogenic genes in mandarin. (DOCX 17 kb)

## Abbreviations

ANS: Anthocyanidin synthase
BCH1: Beta-carotene 3-hydroxylase 1
BCH3: Beta-carotene 3-hydroxylase 3
CCD1: Carotenoid cleavage dioxygenase 1
CHS: Chalcone synthase
COG: The clusters of orthologous groups of proteins database
CYCB: Capsorubin synthase
DFR: Dihydroflavonol 4-reductase
GATK: Genome Analysis Toolkit
GBS: Genotyping-by-sequencing
GO: Gene Ontology enrichment database
HJ: Hujingmilu
KEGG: The Kyoto encyclopedia of genes and genomes database
LCYB: Lycopeneβ-cyclase
NCBI: National center for biotechnology information
NGS: Next-generation sequencing
Nr: NCBI non-redundant database
PK: Ponkan
PSY1: Phytoene synthase 1
PSY2: Phytoene synthase 2
RNA-Seq: RNA-sequencing
SNP: Single nucleotide polymorphisms
Swiss-Prot: Swiss-Prot protein database
UFGT: UDP-glucose: flavonoid 3-*O*-glucosyltransfersae
VDE: Violaxanthin de-epoxidase
YL: Yulu
YP: Yellowish-peeled Ponkan
ZEP: Zeaxanthin epoxidase
