# Supplementary material for: A high-throughput SNP discovery strategy for RNA-seq data
Source: BMC Genomics. 2019 Feb 27;20:160. doi: 10.1186/s12864-019-5533-4 (PMC6391812; doi:10.1186/s12864-019-5533-4)
Supplement: Supplementary file 1 — Table S1. Assembly statistics of peach and mandarin transcriptomes under Trinity. (DOCX 17 kb) [file 12864_2019_5533_MOESM1_ESM.docx]

**Additional File 1: Table S1. Assembly statistics of peach and mandarin transcriptomes under Trinity.**

| **Plant-Paired-end read length →** | **peach-125bp** | | **peach-150bp** | | | **mandarin-150bp** | | |
| --- | --- | --- | --- | --- | --- | --- | --- | --- |
| **Length ↓** | **Transcripts** | **Unigenes** | | **Transcripts** | **Unigenes** | | **Transcripts** | **Unigenes** |
| **200-300** | 16139(15.14%) | 14448(32.49%) | | 19514(18.88%) | 16845(34.21%) | | 28563(17.04%) | 24691(37.33%) |
| **300-500** | 12795(12.01%) | 9755(21.94%) | | 14767(14.29%) | 10747(21.83%) | | 21925(13.08%) | 15985(24.17%) |
| **500-1000** | 16403(15.39%) | 7468(16.79%) | | 17037(16.49%) | 8509(17.28%) | | 23458(14.00%) | 11061(16.72%) |
| **1000-2000** | 27502(25.81%) | 6894(15.50%) | | 25724(24.89%) | 7427(15.08%) | | 37933(22.64%) | 7675(11.60%) |
| **2000+** | 33735(31.65%) | 5905(13.28%) | | 26300(25.45%) | 5707(11.59%) | | 55701(33.24%) | 6736(10.18%) |
| **Total Number** | 106574 | 44470 | | 103342 | 49235 | | 167580 | 66148 |
| **Total Length** | 1.72E+08 | 40888043 | | 1.43E+08 | 42440319 | | 2.73E+08 | 52181352 |
| **N50 Length** | 2521 | 1807 | | 2252 | 1663 | | 2631 | 1587 |
| **Mean Length** | 1618.19 | 919.45 | | 1386.36 | 861.99 | | 1630.85 | 788.86 |
