# Supplementary material for: A high-throughput SNP discovery strategy for RNA-seq data
Source: BMC Genomics. 2019 Feb 27;20:160. doi: 10.1186/s12864-019-5533-4 (PMC6391812; doi:10.1186/s12864-019-5533-4)
Supplement: Supplementary file 2 — Table S2. Summary of read mapping of peach (cv. HJ and cv. YL) and mandarin (cv. PK and cv. YP) transcriptomes under Trinity. (DOCX 18 kb) [file 12864_2019_5533_MOESM2_ESM.docx]

**Additional File 2: Table S2. Summary of read mapping of peach (cv. HJ and cv. YL) and mandarin transcriptomes under Trinity.**

| Plant-Paired-end read length | HJ-125bp | YL-125bp | HJ-150bp | YL-150bp | PK-150bp | YP-150bp |
| --- | --- | --- | --- | --- | --- | --- |
| Total Reads  (Percentage) | 22,182,670  (100%) | 20,122,397  (100%) | 25,905,927  (100%) | 21,836,682  (100%) | 29,498,009  (100%) | 23,641,003  (100%) |
| Mapped Reads  (Percentage) | 18,590,136  (83.80%) | 16,762,622  (83.31%) | 20,249,484  (78.17%) | 16,663,198  (76.31%) | 23,194,883  (78.63%) | 18,846,492  (79.72%) |
| Uniq-mapped Reads  (Percentage) | 7,444,846  (40.05%) | 6,901,854  (41.18%) | 14,627,026  (72.23%) | 11,821,065  (70.94%) | 13,414,569  (57.83%) | 10,752,750  (57.05%) |
| Multi-mappd Reads  (Percentage) | 11,145,291  (59.95%) | 9,860,768  (58.82%) | 5,622,458  (27.77%) | 4,842,133  (29.06%) | 9,780,314  (42.17%) | 8,093,742  (42.95%) |
