# Supplementary material for: A high-throughput SNP discovery strategy for RNA-seq data
Source: BMC Genomics. 2019 Feb 27;20:160. doi: 10.1186/s12864-019-5533-4 (PMC6391812; doi:10.1186/s12864-019-5533-4)
Supplement: Supplementary file 3 — Table S3. Number of SNPs predicted from RNA-seq data under different paired-end read lengths, assemblers and SNP callers. The number is the average of two cultivars. (DOCX 17 kb) [file 12864_2019_5533_MOESM3_ESM.docx]

**Additional File 3: Table S3. Number of SNPs predicted from RNA-seq data under different paired-end read lengths, assemblers and SNP callers.** The number is the average of two cultivars.

| **Plant-Paired-end read length** | **Assembler** | **SNP caller** | |
| --- | --- | --- | --- |
|  |  | **GATK** | **GBS** |
| **peach-125bp** | Trinity | 52449 | 39705 |
|  | IDBA_tran | 26931 | 9198 |
|  | oases | 37336 | 10812 |
|  | SOAPdenovo | 40198 | 17380 |
|  | Trans-abyss | 56271 | 20883 |
| **peach-150bp** | Trinity | 33052 | 14002 |
|  | IDBA_tran | 23281 | 7752 |
|  | oases | 40538 | 14658 |
|  | SOAPdenovo | 27428 | 11526 |
|  | Trans-abyss | 37126 | 11209 |
| **mandarin-150bp** | Trinity | 118361 | 100030 |
|  | IDBA_tran | 68564 | 43944 |
|  | oases | 106952 | 61562 |
|  | SOAPdenovo | 91649 | 71294 |
|  | Trans-abyss | 127201 | 87689 |
