# Supplementary material for: A high-throughput SNP discovery strategy for RNA-seq data
Source: BMC Genomics. 2019 Feb 27;20:160. doi: 10.1186/s12864-019-5533-4 (PMC6391812; doi:10.1186/s12864-019-5533-4)
Supplement: Supplementary file 4 — Table S4. The detailed information of 40 authentic SNPs in five anthocyanin biosynthesis related genes in peach. (DOCX 17 kb) [file 12864_2019_5533_MOESM4_ESM.docx]

**Additional File 4: Table S4. The detailed information of 40 authentic SNPs in five anthocyanin biosynthesis related genes in peach.**

| **Gene** | **#Chr (Trinity)** | **Pos** | **HJ (Ref/Alt)** | **YL (Ref/Alt)** |
| --- | --- | --- | --- | --- |
| **CHS** | c27590.graph_c0 | 932 | C/T | C/T |
|  | c27590.graph_c0 | 1114 | C/T | C/T |
| **DFR** | c12272.graph_c0 | 1040 | G/G | G/A |
|  | c12272.graph_c0 | 1327 | A/A | A/G |
| **ANS** | c6373.graph_c0 | 1630 | G/A | G/A |
| **UFGT** | c14205.graph_c0 | 339 | A/C | A/A |
|  | c14205.graph_c0 | 919 | G/T | G/G |
|  | c14205.graph_c0 | 1571 | G/G | A/G |
| **WD40-1** | c23790.graph_c0 | 92 | T/G | T/T |
|  | c23790.graph_c0 | 169 | A/C | A/A |
|  | c23790.graph_c0 | 358 | A/C | A/C |
|  | c23790.graph_c0 | 437 | A/C | A/A |
|  | c23790.graph_c0 | 445 | T/T | T/A |
|  | c23790.graph_c0 | 817 | T/T | T/C |
|  | c23790.graph_c0 | 967 | G/A | G/A |
|  | c23790.graph_c0 | 982 | A/A | A/G |
|  | c23790.graph_c0 | 1055 | A/T | A/T |
|  | c23790.graph_c0 | 1095 | C/A | C/A |
|  | c23790.graph_c0 | 2006 | A/A | A/G |
|  | c23790.graph_c0 | 2627 | G/G | G/C |
