# Supplementary material for: A high-throughput SNP discovery strategy for RNA-seq data
Source: BMC Genomics. 2019 Feb 27;20:160. doi: 10.1186/s12864-019-5533-4 (PMC6391812; doi:10.1186/s12864-019-5533-4)
Supplement: Supplementary file 5 — Table S5. The detailed information of 240 authentic SNPs in nine carotenogenic genes in mandarin. (DOCX 21 kb) [file 12864_2019_5533_MOESM5_ESM.docx]

**Additional File 5: Table S5. The detailed information of 240 authentic SNPs in nine carotenogenic genes in mandarin.**

| **Gene** | **#Chr (Trinity)** | **Pos** | **PK (Ref/Alt)** | **YP (Ref/Alt)** |
| --- | --- | --- | --- | --- |
| **ZEP** | c40549.graph_c0 | 3637 | T/C | T/C |
|  | c40549.graph_c0 | 4439 | G/T | G/T |
|  | c40549.graph_c0 | 5383 | G/A | G/A |
|  | c40549.graph_c0 | 5384 | G/A | G/A |
|  | c40549.graph_c0 | 6196 | T/C | T/C |
| **PSY1** | c38711.graph_c0 | 222 | G/C | G/G |
|  | c38711.graph_c0 | 254 | G/A | G/A |
|  | c38711.graph_c0 | 357 | C/G | C/G |
|  | c38711.graph_c0 | 389 | A/T | A/T |
|  | c38711.graph_c0 | 434 | A/G | A/G |
|  | c38711.graph_c0 | 442 | G/A | G/A |
|  | c38711.graph_c0 | 600 | A/G | A/G |
|  | c38711.graph_c0 | 612 | A/G | A/G |
|  | c38711.graph_c0 | 929 | T/C | T/C |
|  | c38711.graph_c0 | 1027 | C/T | C/T |
|  | c38711.graph_c0 | 1033 | C/A | C/A |
|  | c38711.graph_c0 | 1095 | C/G | C/G |
|  | c38711.graph_c0 | 1198 | C/T | C/T |
|  | c38711.graph_c0 | 1377 | G/A | G/A |
|  | c38711.graph_c0 | 1420 | A/G | A/G |
|  | c38711.graph_c0 | 1453 | A/G | A/G |
|  | c38711.graph_c0 | 1459 | T/C | T/C |
|  | c38711.graph_c0 | 1461 | A/T | A/T |
|  | c38711.graph_c0 | 1556 | C/A | C/A |
|  | c38711.graph_c0 | 1714 | A/G | A/G |
|  | c38711.graph_c0 | 1800 | A/C | A/C |
|  | c38711.graph_c0 | 1901 | A/G | A/G |
|  | c38711.graph_c0 | 1931 | T/A | T/A |
|  | c38711.graph_c0 | 2038 | G/A | G/G |
| **PSY2** | c31999.graph_c0 | 242 | A/C | A/C |
|  | c31999.graph_c0 | 312 | G/A | G/A |
|  | c31999.graph_c0 | 586 | T/C | T/C |
|  | c31999.graph_c0 | 655 | A/G | A/G |
|  | c31999.graph_c0 | 673 | A/T | A/T |
|  | c31999.graph_c0 | 856 | C/T | C/T |
|  | c31999.graph_c0 | 862 | A/G | A/G |
| **BCH1** | c18724.graph_c0 | 121 | A/T | A/T |
|  | c18724.graph_c0 | 505 | C/A | C/A |
|  | c18724.graph_c0 | 631 | G/A | G/A |
|  | c18724.graph_c0 | 121 | A/T | A/T |
| **BCH3** | c32562.graph_c0 | 948 | A/C | A/C |
|  | c32562.graph_c0 | 2064 | A/G | A/G |
| **VDE** | c39612.graph_c1 | 239 | T/C | T/C |
|  | c39612.graph_c1 | 273 | C/T | C/T |
|  | c39612.graph_c1 | 332 | A/G | A/G |
|  | c39612.graph_c1 | 559 | C/T | C/T |
|  | c39612.graph_c1 | 616 | G/A | G/A |
|  | c39612.graph_c1 | 943 | A/C | A/C |
|  | c39612.graph_c1 | 1040 | G/A | G/A |
|  | c39612.graph_c1 | 1050 | G/A | G/A |
|  | c39612.graph_c1 | 1120 | T/G | T/G |
|  | c39612.graph_c1 | 1194 | T/T | T/A |
|  | c39612.graph_c1 | 1218 | C/T | C/T |
|  | c39612.graph_c1 | 1260 | T/C | T/C |
|  | c39612.graph_c1 | 1482 | A/G | A/G |
|  | c39612.graph_c1 | 1578 | A/T | A/T |
|  | c39612.graph_c1 | 1659 | T/C | T/C |
|  | c39612.graph_c1 | 1696 | T/A | T/A |
|  | c39612.graph_c1 | 1722 | T/A | T/A |
|  | c39612.graph_c1 | 1732 | A/G | A/G |
| **LCYB** | c40868.graph_c0 | 43 | T/A | T/A |
|  | c40868.graph_c0 | 155 | T/C | T/C |
|  | c40868.graph_c0 | 192 | T/A | T/A |
|  | c40868.graph_c0 | 194 | C/T | C/T |
|  | c40868.graph_c0 | 243 | G/T | G/T |
|  | c40868.graph_c0 | 382 | T/G | T/G |
|  | c40868.graph_c0 | 422 | G/A | G/A |
|  | c40868.graph_c0 | 518 | C/T | C/T |
|  | c40868.graph_c0 | 524 | G/A | G/A |
|  | c40868.graph_c0 | 569 | A/G | A/G |
|  | c40868.graph_c0 | 723 | A/G | A/G |
|  | c40868.graph_c0 | 737 | G/C | G/C |
|  | c40868.graph_c0 | 803 | C/T | C/T |
|  | c40868.graph_c0 | 908 | A/G | A/G |
|  | c40868.graph_c0 | 926 | G/C | G/C |
|  | c40868.graph_c0 | 938 | A/G | A/G |
|  | c40868.graph_c0 | 1228 | A/G | A/G |
|  | c40868.graph_c0 | 1247 | A/G | A/G |
|  | c40868.graph_c0 | 1580 | C/T | C/T |
|  | c40868.graph_c0 | 1697 | A/C | A/C |
|  | c40868.graph_c0 | 1757 | C/T | C/T |
|  | c40868.graph_c0 | 1762 | T/G | T/G |
|  | c40868.graph_c0 | 1863 | T/A | T/A |
|  | c40868.graph_c0 | 2009 | T/G | T/G |
|  | c40868.graph_c0 | 2052 | A/G | A/G |
|  | c40868.graph_c0 | 2079 | A/G | A/G |
|  | c40868.graph_c0 | 2209 | T/A | T/A |
|  | c40868.graph_c0 | 2235 | A/G | A/G |
|  | c40868.graph_c0 | 2252 | A/G | A/G |
|  | c40868.graph_c0 | 2306 | T/C | T/C |
| **CYCB** | c36565.graph_c0 | 349 | A/C | A/C |
|  | c36565.graph_c0 | 364 | A/G | A/G |
|  | c36565.graph_c0 | 367 | A/C | A/C |
|  | c36565.graph_c0 | 379 | G/C | G/C |
|  | c36565.graph_c0 | 406 | A/G | A/G |
|  | c36565.graph_c0 | 426 | A/G | A/G |
|  | c36565.graph_c0 | 429 | A/G | A/G |
|  | c36565.graph_c0 | 438 | A/G | A/G |
|  | c36565.graph_c0 | 502 | C/G | C/G |
|  | c36565.graph_c0 | 507 | T/A | T/A |
|  | c36565.graph_c0 | 532 | A/G | A/G |
|  | c36565.graph_c0 | 710 | G/A | G/A |
|  | c36565.graph_c0 | 843 | T/C | T/C |
|  | c36565.graph_c0 | 850 | C/A | C/A |
|  | c36565.graph_c0 | 855 | G/A | G/A |
|  | c36565.graph_c0 | 925 | G/A | G/A |
|  | c36565.graph_c0 | 985 | C/G | C/G |
|  | c36565.graph_c0 | 992 | A/G | A/G |
|  | c36565.graph_c0 | 1367 | G/A | G/A |
|  | c36565.graph_c0 | 1808 | C/T | C/T |
|  | c36565.graph_c0 | 1839 | T/C | T/C |
|  | c36565.graph_c0 | 1898 | G/C | G/C |
|  | c36565.graph_c0 | 1983 | G/A | G/A |
|  | c36565.graph_c0 | 2033 | T/T | T/C |
| **CCD1** | c33455.graph_c0 | 194 | C/A | C/A |
|  | c33455.graph_c0 | 487 | T/A | T/A |
|  | c33455.graph_c0 | 611 | C/T | C/T |
|  | c33455.graph_c0 | 740 | T/C | T/C |
|  | c33455.graph_c0 | 763 | A/G | A/G |
|  | c33455.graph_c0 | 1460 | C/T | C/T |
|  | c33455.graph_c0 | 1967 | G/C | G/C |
