# Supplementary material for: A high-throughput SNP discovery strategy for RNA-seq data
Source: BMC Genomics. 2019 Feb 27;20:160. doi: 10.1186/s12864-019-5533-4 (PMC6391812; doi:10.1186/s12864-019-5533-4)
Supplement: Supplementary file 7 — Table S7. An overview of the number of SNPs predicted in targeted genes from peach with ten different strategies under the read length of 150 bp. Values in brackets denote the ratio of heterozygous and homozygous SNP (HZ:HM). (DOCX 19 kb) [file 12864_2019_5533_MOESM7_ESM.docx]

**Additional File 7: Table S7. An overview of the number of SNPs predicted in targeted genes from peach with ten different strategies under the read length of 150 bp.** Values in brackets denote the ratio of heterozygous and homozygous SNP (HZ:HM).

| **Gene** | **Authentic**  **SNP** | | **Trinity** | | | | **IDBA_tran** | | | | **oases** | | | | **SOAPdenovo** | | | | **trans-abyss** | | | |
| --- | --- | --- | --- | --- | --- | --- | --- | --- | --- | --- | --- | --- | --- | --- | --- | --- | --- | --- | --- | --- | --- | --- |
|  |  |  | **GATK** | | **GBS** | | **GATK** | | **GBS** | | **GATK** | | **GBS** | | **GATK** | | **GBS** | | **GATK** | | **GBS** | |
|  | **HJ** | **YL** | **HJ** | **YL** | **HJ** | **YL** | **HJ** | **YL** | **HJ** | **YL** | **HJ** | **YL** | **HJ** | **YL** | **HJ** | **YL** | **HJ** | **YL** | **HJ** | **YL** | **HJ** | **YL** |
| **CHS** | **2**  **(2：0)** | **2**  **(2：0)** | **2**  **(2：0)** | **2**  **(2：0)** | **0** | **0** | **1**  **(1：0)** | **1**  **(1：0)** | **1**  **(1：0)** | **1**  **(1：0)** | **0** | **0** | **0** | **0** | **0** | **0** | **0** | **0** | **3**  **(2：1)** | **3**  **(1：2)** | **0** | **0** |
| **DFR** | **2**  **(0：2)** | **2**  **(2：0)** | **2**  **(0：2)** | **2**  **(2：0)** | **0** | **0** | **3**  **(1：2)** | **3**  **(3：0)** | **0** | **0** | **2**  **(0：2)** | **2**  **(2：0)** | **0** | **0** | **2**  **(0：2)** | **2**  **(2：0)** | **0** | **0** | **2**  **(0：2)** | **2**  **(2：0)** | **0** | **0** |
| **ANS** | **1**  **(1：0)** | **1**  **(1：0)** | **1**  **(1：0)** | **1**  **(1：0)** | **0** | **0** | **0** | **0** | **0** | **0** | **4**  **(1：3)** | **4**  **(1：3)** | **0** | **0** | **1**  **(1：0)** | **1**  **(1：0)** | **0** | **0** | **2**  **(1：1)** | **2**  **(1：1)** | **0** | **0** |
| **UFGT** | **3**  **(2：1)** | **3**  **(1：2)** | **3**  **(2：1)** | **3**  **(1：2)** | **0** | **0** | **0** | **0** | **0** | **0** | **5**  **(4：1)** | **5**  **(0：5)** | **0** | **0** | **5**  **(2：3)** | **5**  **(3：2)** | **0** | **0** | **0** | **0** | **0** | **0** |
| **WD40** | **12**  **(7：5)** | **12**  **(9：3)** | **12**  **(7：5)** | **12**  **(9：3)** | **3**  **(3：0)** | **3**  **(3：0)** | **12**  **(7：5)** | **12**  **(9：3)** | **3**  **(3：0)** | **3**  **(3：0)** | **9**  **(4：5)** | **9**  **(8：1)** | **3**  **(3：0)** | **3**  **(3：0)** | **11**  **(6：5)** | **11**  **(9：2)** | **3**  **(3：0)** | **3**  **(3：0)** | **12**  **(7：5)** | **12**  **(8：4)** | **1**  **(1：0)** | **1**  **(1：0)** |
