# Supplementary material for: A high-throughput SNP discovery strategy for RNA-seq data
Source: BMC Genomics. 2019 Feb 27;20:160. doi: 10.1186/s12864-019-5533-4 (PMC6391812; doi:10.1186/s12864-019-5533-4)
Supplement: Supplementary file 8 — Table S8. An overview of the number of SNPs predicted in targeted genes from mandarin with ten different strategies under the read length of 150 bp. Values in brackets denote the ratio of heterozygous and homozygous SNP (HZ:HM). (DOCX 22 kb) [file 12864_2019_5533_MOESM8_ESM.docx]

**Additional File 8: Table S8. An overview of the number of SNPs predicted in targeted genes from mandarin with ten different strategies under the read length of 150 bp.** Values in brackets denote the ratio of heterozygous and homozygous SNP (HZ:HM).

| **Gene** | **Authentic**  **SNP** | | **Trinity** | | | | **IDBA_tran** | | | | **oases** | | | | **SOAPdenovo** | | | | **trans-abyss** | | | |
| --- | --- | --- | --- | --- | --- | --- | --- | --- | --- | --- | --- | --- | --- | --- | --- | --- | --- | --- | --- | --- | --- | --- |
|  |  |  | **GATK** | | **GBS** | | **GATK** | | **GBS** | | **GATK** | | **GBS** | | **GATK** | | **GBS** | | **GATK** | | **GBS** | |
|  | **PK** | **YP** | **PK** | **YP** | **PK** | **YP** | **PK** | **YP** | **PK** | **YP** | **PK** | **YP** | **PK** | **YP** | **PK** | **YP** | **PK** | **YP** | **PK** | **YP** | **PK** | **YP** |
| **ZEP** | **5**  **(5：0)** | **5**  **(5：0)** | **5**  **(5：0)** | **5**  **(5：0)** | **3**  **(3：0)** | **3**  **(3：0)** | **0** | **0** | **0** | **0** | **0** | **0** | **0** | **0** | **0** | **0** | **1**  **(1：0)** | **1**  **(1：0)** | **3**  **(3：0)** | **3**  **(3：0)** | **4**  **(4：0)** | **4**  **(4：0)** |
| **PSY1** | **24**  **(24：0)** | **24**  **(22：2)** | **24**  **(24：0)** | **24**  **(22：2)** | **3**  **(3：0)** | **3**  **(3：0)** | **3**  **(3：0)** | **3**  **(3：0)** | **4**  **(4：0)** | **4**  **(4：0)** | **9**  **(7：2)** | **9**  **(7：2)** | **6**  **(6：0)** | **6**  **(6：0)** | **8**  **(8：0)** | **8**  **(7：1)** | **6**  **(6：0)** | **6**  **(6：0)** | **22**  **(22：0)** | **22**  **(17：5)** | **4**  **(4：0)** | **4**  **(4：0)** |
| **PSY2** | **7**  **(7：0)** | **7**  **(7：0)** | **7**  **(7：0)** | **7**  **(7：0)** | **7**  **(7：0)** | **7**  **(7：0)** | **0** | **0** | **0** | **0** | **8**  **(7：1)** | **8**  **(7：1)** | **8**  **(8：0)** | **8**  **(8：0)** | **6**  **(6：0)** | **6**  **(6：0)** | **9**  **(9：0)** | **9**  **(9：0)** | **7**  **(7：0)** | **7**  **(7：0)** | **7**  **(7：0)** | **7**  **(7：0)** |
| **BCH1** | **3**  **(3：0)** | **3**  **(3：0)** | **3**  **(3：0)** | **3**  **(3：0)** | **1**  **(1：0)** | **1**  **(1：0)** | **4**  **(4：0)** | **4**  **(3：1)** | **4**  **(4：0)** | **4**  **(4：0)** | **10**  **(5：5)** | **10**  **(7：3)** | **7**  **(7：0)** | **7**  **(7：0)** | **3**  **(3：0)** | **3**  **(3：0)** | **1**  **(1：0)** | **1**  **(1：0)** | **3**  **(3：0)** | **3**  **(3：0)** | **1**  **(1：0)** | **1**  **(1：0)** |
| **BCH3** | **2**  **(2：0)** | **2**  **(2：0)** | **2**  **(2：0)** | **2**  **(2：0)** | **2**  **(2：0)** | **2**  **(2：0)** | **3**  **(2：1)** | **3**  **(1：2)** | **0** | **0** | **6**  **(1：5)** | **6**  **(3：3)** | **1**  **(1：0)** | **1**  **(1：0)** | **2**  **(2：0)** | **2**  **(2：0)** | **2**  **(2：0)** | **2**  **(2：0)** | **1**  **(0：1)** | **1**  **(0：1)** | **0** | **0** |
| **VDE** | **18**  **(17：1)** | **18**  **(18：0)** | **18**  **(17：1)** | **18**  **(18：0)** | **17**  **(17：0)** | **17**  **(17：0)** | **0** | **0** | **0** | **0** | **5**  **(5：0)** | **5**  **(5：0)** | **5**  **(5：0)** | **5**  **(5：0)** | **5**  **(4：1)** | **5**  **(4：1)** | **0** | **0** | **18**  **(17：1)** | **18**  **(17：1)** | **17**  **(17：0)** | **17**  **(17：0)** |
| **LCYB** | **30**  **(30：0)** | **30**  **(30：0)** | **30**  **(30：0)** | **30**  **(30：0)** | **28**  **(28：0)** | **28**  **(28：0)** | **0** | **0** | **0** | **0** | **6**  **(6：0)** | **6**  **(3：3)** | **0** | **0** | **27**  **(27：0)** | **27**  **(27：0)** | **25**  **(25：0)** | **25**  **(25：0)** | **32**  **(27：5)** | **32**  **(27：5)** | **16**  **(16：0)** | **16**  **(16：0)** |
| **CYCB** | **24**  **(23：1)** | **24**  **(24：0)** | **24**  **(23：1)** | **24**  **(24：0)** | **0** | **0** | **0** | **0** | **0** | **0** | **0** | **0** | **0** | **0** | **11**  **(10：1)** | **11**  **(11：0)** | **1**  **(1：0)** | **1**  **(1：0)** | **10**  **(9：1)** | **10**  **(10：0)** | **0** | **0** |
| **CCD1** | **7**  **(7：0)** | **7**  **(7：0)** | **7**  **(7：0)** | **7**  **(7：0)** | **0** | **0** | **7**  **(4：3)** | **7**  **(4：3)** | **5**  **(5：0)** | **5**  **(5：0)** | **23**  **(12：11)** | **23**  **(10：13)** | **0** | **0** | **6**  **(6：0)** | **6**  **(5：1)** | **6**  **(6：0)** | **6**  **(6：0)** | **7**  **(7：0)** | **7**  **(7：0)** | **1**  **(1：0)** | **1**  **(1：0)** |
