# Supplementary figures and images for: A high-throughput SNP discovery strategy for RNA-seq data
Source: BMC Genomics. 2019 Feb 27;20:160. doi: 10.1186/s12864-019-5533-4 (PMC6391812; doi:10.1186/s12864-019-5533-4)

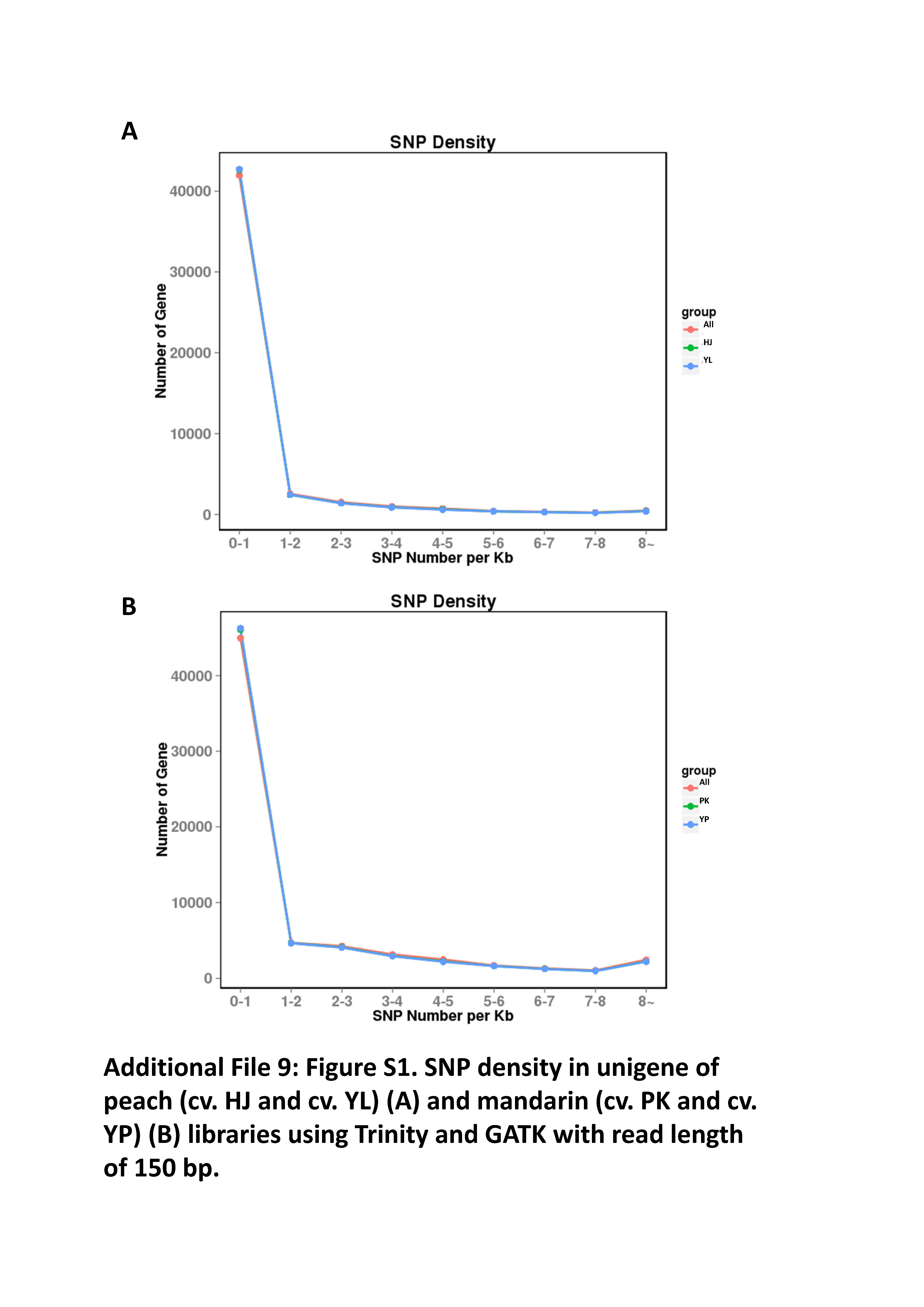

Supplement: Supplementary file 9 — Figure S1. SNP density in unigene of peach (cv. HJ and cv. YL) (A) and mandarin (cv. PK and cv. YP) (B) libraries using Trinity and GATK with read length of 150 bp. (JPG 1491 kb) [file 12864_2019_5533_MOESM9_ESM.jpg]

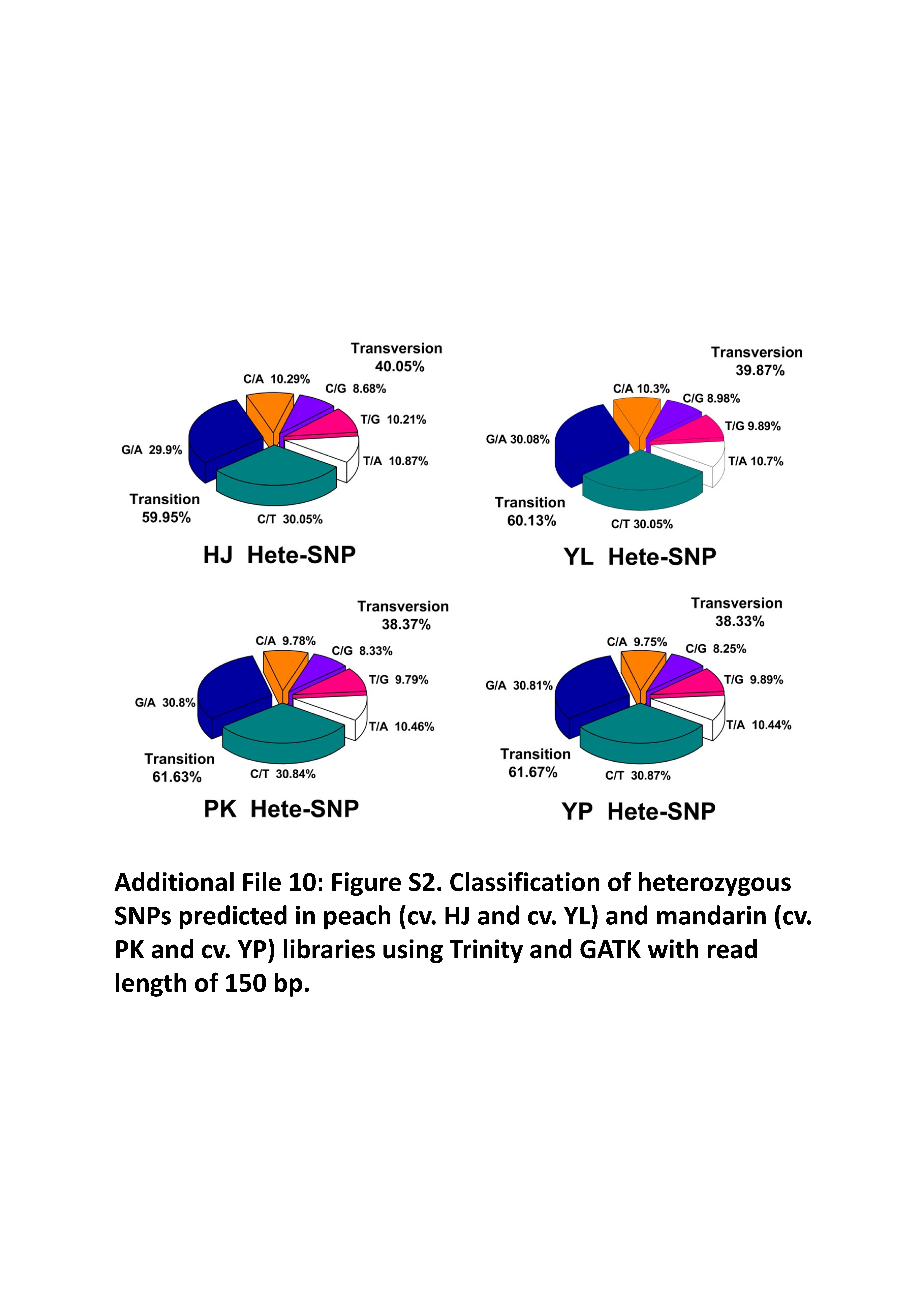

Supplement: Supplementary file 10 — Figure S2. Classification of heterozygous SNPs predicted in peach (cv. HJ and cv. YL) and mandarin (cv. PK and cv. YP) libraries using Trinity and GATK with read length of 150 bp. (JPG 1763 kb) [file 12864_2019_5533_MOESM10_ESM.jpg]
