# Supplementary material for: A high-throughput SNP discovery strategy for RNA-seq data
Source: BMC Genomics. 2019 Feb 27;20:160. doi: 10.1186/s12864-019-5533-4 (PMC6391812; doi:10.1186/s12864-019-5533-4)
Supplement: Supplementary file 11 — Table S9. Primers used for PCR amplification of five anthocyanin biosynthesis related genes in peach and nine carotenogenic genes in mandarin. (DOCX 17 kb) [file 12864_2019_5533_MOESM11_ESM.docx]

**Additional File 11: Table S9. Primers used for PCR amplification of five anthocyanin biosynthesis related genes in peach and nine carotenogenic genes in mandarin.**

| **Gene** | **Forward primer (5′ to 3′ )** | **Reverse primer (5′ to 3′ )** | **GenBank Accession** |
| --- | --- | --- | --- |
| ***PpCHS*** | GTTGAAAATGGTGACCGTGGAG | CAAGCATTCAAGCATTCAAGCC | Prupe.1G002900 |
| ***PpDFR*** | GAATCTGTTTGTGTGACGGGC | CAAACATTTCATGCTCGTTCAAC | Prupe.1G376400 |
| ***PpANS*** | GCACTGTTTGAAACTCTGAAAGGAC | CTAGAAGAGCAGATACATGGCAACC | Prupe.5G086700 |
| ***PpUFGT*** | CCCCACTCCTTTGCCACTATC | GCTTTTAGTTGGATTTTATTGAGGG | Prupe.2G324700 |
| ***PpWD40-1*** | CCTCTGCTTCATCCTCTCAACC | CTAGAACCCCAATGAGAGTACCG | Prupe.2G319500 |
| ***CitZEP*** | TCCAGCACCAGTACCAACATCAGAA | CAATGCGTCATCGTCACGAAACC | Cs7g13880 |
| ***CitPSY1*** | TGCCAACCATAGCAATAGCAATAGC | CAGGGTTACTTGTACAGGCAATTCA | Cs6g15910 |
| ***CitPSY2*** | ACATGGTCTGCTGATTGAATGCTTG | CCACAGCTCGATGCTATGTTTCTTG | orange1.1t02108 |
| ***CitBCH1*** | TTAGAACCATTTAGAGGAGTTACCA | CATTTGCTCTCTCTGTTGGTGC | Cs9g19270 |
| ***CitBCH3*** | TAGTCCCAGCATTGCATCAAAAGTA | GATTCAAGGCATGTTCTCTTCGTGT | orange1.1t01058 |
| ***CitVDE*** | CACACAGTTGCCTTGGCGCC | TTTTGGAAAATACAGAGGAAAGATA | Cs5g26080 |
| ***CitLCYB*** | GCAACACAAGCTTCATCTTTACCAA | GTATCACAAGTGTTGGTCTTAGTCA | orange1.1t00772 |
| ***CitCYCB*** | GTATATGGTGCGGTGGCAGCAGTAG | AATTTCTGGCTTCGCCCCTGG | XM_006424132.2 |
| ***CitCCD1*** | TAAGTACATCCGTTTTAGCCAATCG | AAATATTATCTTTTGCTGTGGGTCG | Cs7g01710 |
